# Evaluation of Betacoronavirus OC43 and SARS-CoV-2 Elimination by Zefero Air Sanitizer Device in a Novel Laboratory Recirculation System

**DOI:** 10.3390/pathogens11020221

**Published:** 2022-02-08

**Authors:** Marco Sebastiano Nicolò, Maria Giovanna Rizzo, Nicoletta Palermo, Concetta Gugliandolo, Salvatore Cuzzocrea, Salvatore P. P. Guglielmino

**Affiliations:** Department of Chemical, Biological, Pharmaceutical and Environmental Sciences, University of Messina, V.le F. Stagno D’Alcontres, 31, 98166 Messina, Italy; mnicolo@unime.it (M.S.N.); mariagiovanna.rizzo@unime.it (M.G.R.); nicopal31@gmail.com (N.P.); cgugliandolo@unime.it (C.G.); salvatore.cuzzocrea@unime.it (S.C.)

**Keywords:** airborne disease viruses, OC43, SARS-CoV-2, virus decontamination, air sanitization

## Abstract

Indoor air sanitizers contrast airborne diseases and particularly severe acute respiratory syndrome coronavirus 2 (SARS-CoV-2)/Coronavirus disease 2019 (COVID-19). The commercial air sanitizer Zefero (Cf7 S.r.l., San Giovanni La Punta, Italy) works alternatively using a set of integrated disinfecting technologies (namely Photocatalysis/UV mode) or by generating ozone (Ozone mode). Here we evaluated the virucidal efficacy of Zefero setup modes against human Betacoronavirus OC43 and SARS-CoV-2. For this purpose, we designed a laboratory test system in which each virus, as aerosol, was treated with Photocatalysis/UV or Ozone mode and returned into a recirculation plexiglass chamber. Aerosol samples were collected after different times of exposure, corresponding to different volumes of air treated. The viral RNA concentration was determined by qRT-PCR. In Photocatalysis/UV mode, viral RNA of OC43 or SARS-CoV-2 was not detected after 120 or 90 min treatment, respectively, whereas in Ozone mode, viruses were eliminated after 30 or 45 min, respectively. Our results indicated that the integrated technologies used in the air sanitizer Zefero are effective in eliminating both viruses. As a reliable experimental system, the recirculation chamber developed in this study represents a suitable apparatus for effectively comparing the disinfection capacity of different air sanitizers.

## 1. Introduction

The social and economic consequences caused by pandemics of airborne disease viruses are considered the first global concern for public health. In the past decades, severe acute respiratory syndrome (SARS), at its first outbreak in 2002–2003, involved about 8000 people, causing the deaths of 700. SARS spread around 37 countries, mostly in Asia, with an economic loss of USD 18 billion [1]. Middle East Respiratory Syndrome (MERS), caused by the Betacoronavirus MERS-CoV or EMC/2012 (HCoV-EMC/2012), was firstly described in Saudi Arabia in 2012, and outbreaks spread across 27 countries in Europe, North America, the Middle East and Asia, with a total of 2494 people affected and 858 associated deaths (34.4% case–fatality ratio) [2]. In the same way, the most recent flu pandemic was the 2009 swine flu pandemic, which originated in Mexico and resulted in hundreds of thousands of deaths [3], and seasonal influenza in 2017–2018 caused 45 million influenza illnesses and 61,000 influenza-associated deaths in the USA, as reported by CDC [4]. In the present, SARS Coronavirus 2 (SARS-CoV-2), the novel homologous strain of SARS-CoV-1, caused the coronavirus disease 2019 (COVID-19) pandemics, which started in December 2019 at Wuhan (China) among a cluster of patients affected by an unidentified form of viral pneumonia [2,5]. Within 1 month of the first identification, the virus spread all over the world, and, in March 2020, WHO declared COVID-19 as a pandemic [6]. The WHO reported that more than 5,411,759 people died all around the world up to December 2021 [7].

In order to contrast all airborne diseases spreading and particularly COVID-19, one important strategy is mainly focused on the safety of indoor environments using both innovative air purification procedures and the setup of indoor air cleaning systems. Several technologies are employed in air sanitization. Among them, the most considered are non-thermal plasma (NTP), ultraviolet (UV) light, use of antimicrobial material-embedded filters, electrical ionization and photocatalytic oxidation (PCO) [8,9]. NTP, also called cold plasma, is generated by the action of electrical discharges within a neutral gas and is formed by ions, electrons and radical species [10]. The efficacy of NTP on airborne microorganisms was also investigated [11,12]. Recent studies showed that plasma acts on purified SARS-CoV-2 RNA, as well as alters the spike S1 protein, then preventing viral adhesion on host cells [13,14,15]. During NTP generation, ozone is also produced, a highly reactive oxygen species and one of the most potent oxidizing agents that have demonstrated the ability to destroy a wide range of microbes, such as bacteriophage MS2 [10] and several other viruses [16,17,18], including SARS-CoV-2 [19]. Several studies showed the inactivation of SARS-CoV-2 after irradiation by UV-C light [20,21,22]. Filtration is a common strategy used to remove particles from the air. In particular, the high-efficiency particulate air (HEPA) filtration technique is a well-known treatment to manage bioaerosols spreading in laboratories and hospitals [23]. In order to improve the antimicrobial performances of a filter, the basic material (activated carbon granules as well as natural or synthetic fibers) can be doped with several compounds to minimize filter clogging and avoid particle release in the environment. For example, silver nanoparticles (AgNPs) are known to be very active biocides, and they were found to be very effective against *Staphylococcus aureus*, *Enterococcus faecalis* and *Escherichia coli* and against extracellular SARS-CoV-2 [24,25]. Air ionization is based on the principle of the so-called “corona discharge”, which is an electrical discharge generated by the air as a consequence of a high voltage electric field, with the emissions of positive and negative ions. Electrical air ionization, simultaneously producing positive and negative air ions, successfully inactivated *aerosolized Staphylococcus epidermidis* [26] and bacteriophage MS2 [27]. PCO is based on semiconductor oxides that, under energy irradiation, generate highly reactive oxygen species (including •OH, H_2_O_2_, H^+^ and •O_2_^−^), participate in photocatalytic degradation [28]. The activity of PCO on SARS-CoV-2 was recently demonstrated [29].

Air sanitizers where filtration is associated with other disinfecting technologies can be considered as more effective tools than the ones based only on filtration to fight airborne infective agents [30,31]. However, despite an extensive body of research about the antimicrobial activity of single disinfecting technologies, accurate knowledge on the efficiency of commercial indoor air sanitizers is still lacking [32].

The commercial air sanitizer Zefero (Cf7 S.r.l., Italy) can alternatively work using a set of integrated disinfecting technologies, i.e., filtration, UV-C light, photocatalytic oxidation, plasma and air ionization (Photocatalysis/UV mode) or by generating ozone (Ozone mode) [33].

In this study, we evaluated the virucidal efficacy of technologies used by the Zefero device (Photocatalysis/UV and Ozone mode) in eliminating the Betacoronavirus OC43 and SARS-CoV-2. We designed a laboratory test system in which OC43 or SARS-CoV-2 aerosol was continuously recirculated into a plexiglass chamber connected to Zefero device under Photocatalysis/UV or Ozone mode, for different times of exposure (from 15 up to 120 min), corresponding to different volumes of air treated (from 85 to 600 m^3^), until virus elimination. After each treatment, viral RNA concentration was determined by qRT-PCR, and viral copies reduction was related to the time of exposure and the volume of recirculated air.

The used recirculation plexiglass chamber made it possible to evaluate the virucidal efficacy of the device in controlled conditions, rather than in real conditions, such as those inside a room, in which the virucidal capacity could be influenced by several factors, including the device positioning, the decay times and the presence of obstacles that can divert the air flows.

## 2. Results

### 2.1. Effects of Zefero Device Operating in Photocatalysis/UV or Ozone Mode on OC43 Virus

After treatment with Photocatalysis/UV or Ozone mode, the viral copies’ reduction in OC43 was evaluated versus the time of exposure and the relative volume of recirculated air (Table 1).

**Figure 1:**
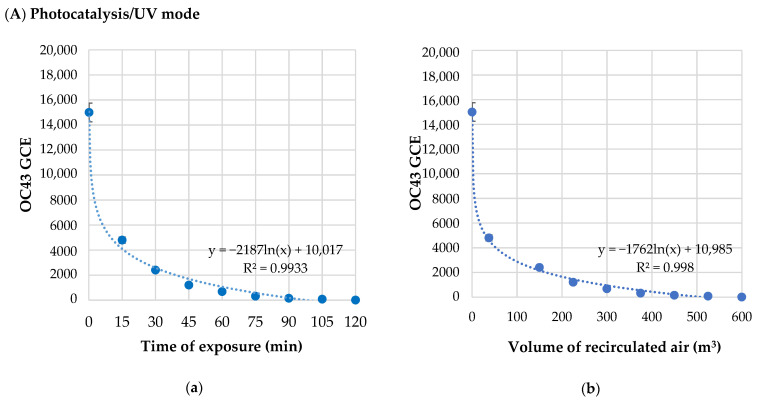

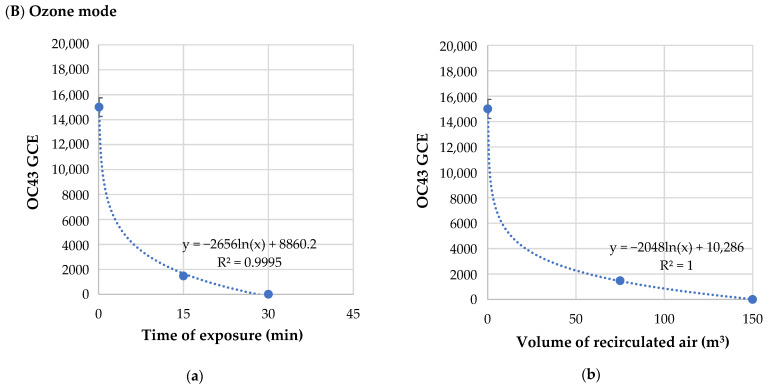
RNA concentration of OC43 virus, expressed as Genome Copy Equivalent (GCE), treated by Photocatalysis/UV (**A**) or Ozone mode (**B**) in relation to the time of exposure (**Aa**,**Ba**) and the volume of recirculated air (**Ab**,**Bb**) in Zefero device.

The OC43 RNA concentration as a function of the time of exposure and the volume of recirculated air under Photocatalysis/UV or Ozone modes are plotted in Figure 1.

In Photocatalysis/UV mode, RNA concentration of OC43 virus was reduced by 90% within 45 min (225 m^3^ of recirculated air), and it was not detectable after 120 min (600 m^3^ of recirculated air). Regression curves showed that the reduction in OC43 GCE had a logarithmic trend (Figure 1) with a strong correlation both between GCE and time of exposure (Figure 1(Aa,Ba)) and volume of recirculated air (Figure 1(Ab,Bb)).

In Ozone mode, RNA concentration of OC43 virus was reduced by 90% after 15 min (75 m^3^ of recirculated air), and it was not detectable after 30 min (150 m^3^ of recirculated air).

The half-life time, calculated as the time needed to halve the genomic RNA concentration of OC43, was 9.62 min after exposure to Photocatalysis/UV mode, whereas it was faster under Ozone mode (4.14 min).

### 2.2. Effects of Zefero Device Operating in Photocatalysis/UV or Ozone Mode on SARS-CoV-2

Viral copies’ reduction in SARS-CoV-2 versus the time of exposure and the relative volume of recirculated air under Photocatalysis/UV or Ozone modes are reported in Table 2.

**Figure 2:**
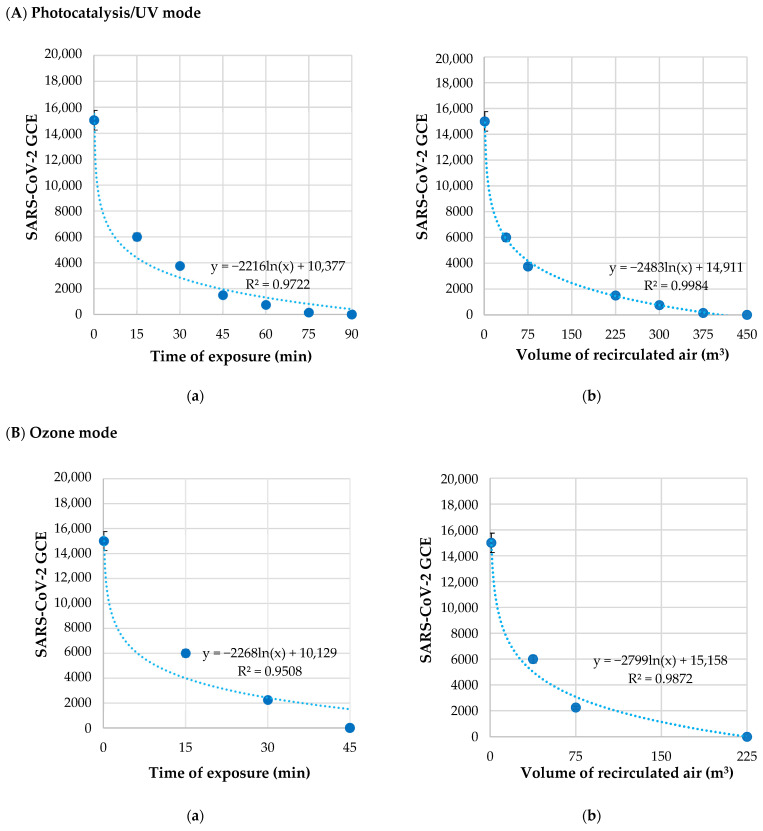
RNA concentration of SARS-CoV-2 virus, expressed as Genome Copy Equivalent (GCE), treated by Photocatalysis/UV (**A**) or Ozone mode (**B**) in relation to the time of exposure (**Aa**,**Ba**) and the volume of recirculated air (**Ab**,**Bb**) in Zefero device.

The SARS-CoV-2 RNA concentration related to the time of exposure and the volume of recirculated air under Photocatalysis/UV or Ozone modes is plotted in Figure 2.

Under Photocatalysis/UV mode (Figure 2A), SARS-CoV-2 GCE was reduced by 90% within 45 min (225 m^3^ of recirculated air), and no viral RNA was detected after 90 min (450 m^3^ of recirculated air).

Under Ozone mode, SARS-CoV-2 concentration was reduced by 85% (75 m^3^ of recirculated air) after 30 min, and the virus was not detected after 45 min (225 m^3^ of recirculated air) (Figure 2B). The reduction in SARS-CoV-2 concentration proceeded according to a logarithmic trend, as showed by regression curves. The half-life time was 12.9 min with Photocatalysis/UV mode, and it was 7.44 min with Ozone mode.

## 3. Discussion

Increasing concerns about the SARS-CoV-2 pandemic have focused global attention on cleaning indoor air and stimulated the development of air purification techniques for disinfecting airborne viruses and bacteria [8]. The United States Environmental Protection Agency (EPA) has outlined that indoor air pollution has to be considered as one of the primary environmental risks to public health because people spend up to 90% of their time in confined places [34] where pollutants tend to accumulate [35]. Moreover, it was suggested that one strategy to prevent or delay the next wave of a pandemic is limiting the virus diffusion when the number of infections grows linearly [36].

Several studies pointed out the resistance of the SARS-CoV-2 virus to aerosols. It was shown that SARS-CoV-2 virions in aerosols could survive for at least 3 h [37]. SARS-CoV-2 RNA was found in aerosol particles with a diameter larger than 1 µm in rooms where patients affected by COVID-19 were hospitalized [38]. Moreover, SARS-CoV-2 RNA was detected in the aerosol at distances of at least 3 m from infected people in indoor environments [25] and in air pollution particles traveling through the air [39].

Then, the intrinsic environmental persistence of SARS-CoV-2 within droplets is a very relevant characteristic that has to be considered when planning strategies to minimize its diffusion.

For this purpose, air sanitizers can be efficient tools in preventing or reducing SARS-CoV-2 spreading in indoor spaces, as well as other airborne pathogenic microbes. Recently, it was demonstrated that a correct spatial dislocation of air sanitizers greatly improves the reduction in airborne infected aerosols [40]. However, very few studies focused on the performances of commercial air sanitizers in eliminating SARS-CoV-2 infected aerosols in indoor spaces [32]. The efficacy of photocatalytic activity against the SARS-CoV-2 virus was demonstrated using industrially coated AgNPs@TiO_2_ ceramic tiles irradiated with UV-A [41]. Several studies demonstrated the virucidal activity of ozone against human respiratory viruses, such as Influenza virus [42], Influenza A and respiratory syncytial virus [43], SARS-CoV-1 [44] and other viruses [45,46,47]. It was recently reported [45] that, depending on ozone concentration and relative humidity, the times required for the virucidal activity ranged from 0.3 to 180 min. A recent study firstly reported that ozone could inactivate 97% of SARS-CoV-2 virus, as dried sample on stainless steel plates, after ozone exposure for 60 min at 1.0 ppm or 100% after 55 min at 6.0 ppm [48]. In another study, SARS-CoV-2 in mucosal sample swabs was fully inactivated by ozone produced by the Bio3gen apparatus (Finlinea s.p.a., Gazzaniga, Bergamo, Italy) with a flow rate of 3.6 L/min and an ozone output of 400 mg/h for a total time of 4 min [49]. Although its high virucidal efficacy, ozone use should be allowed only when no people are present in a given indoor space due to its toxicity.

In this work, we developed a recirculation plexiglass chamber that made it possible to evaluate the effective virucidal capacity of the Zefero device against Betacoronavirus OC43 and SARS-CoV-2 under controlled rather than in real conditions, such as those inside a room, in which virucidal capacity could be influenced by several variables (such as the positioning of the instrument, the decay times, the presence of obstacles that can divert the air flows, etc.). Our results demonstrated that Zefero air sanitizer was effective in eliminating both OC43 and SARS-CoV-2 genomic RNAs under Photocatalysis/UV (after 120 and 90 min, respectively) or Ozone (after 30 or 45 min, respectively) mode. The half-life time, calculated as the time needed to halve the genomic RNA concentration of OC43 or SARS-CoV-2 virus, was 9.62 and 12.9 min with Photocatalysis/UV mode, respectively, and it was faster with Ozone mode (4.14 and 7.44 min, respectively).

However, it is possible that viral infectivity could be lost before the complete destruction of the RNA. It is known that both photocatalysis and ozone significantly damage the viral capsid structures, consequently altering the receptor proteins of the virus.

In conclusion, in this study, the efficacy of technologies used by the commercial air sanitizer Zefero (Photocatalysis/UV and Ozone mode) in eliminating the Betacoranavirus OC43 and SARS-CoV-2 was evaluated by flowing viral aerosols into a recirculation chamber, connected to the air sanitizer device, at different times of exposure. The laboratory test system developed in this study provides a suitable tool for efficiently comparing the disinfecting technologies used in different air sanitizers.

## 4. Materials and Methods

### 4.1. Air Sanitizer Zefero Device: Technical Characteristics

The air sanitizer “Zefero” (Cf7 S.r.l., S. Giovanni La Punta, Catania, Italy) is based on the following technologies: (i) activated carbon filter silvered with potassium permanganate; (ii) double photocatalytic cell, based on a nanotechnological filter in titanium dioxide foam; (iii) 254 nm UV-C lamp; (iv) air ionizer, able to generate anions (200 mln/cm^3^); (v) cold plasma; and (vi) ozonator (5 g h^−1^ of ozone) [33]. The technologies from (i) to (v) act simultaneously when the Photocatalysis/UV mode is switched on, whereas ozonator can be activated independently by selecting the Ozone mode in the device. The device dimensions are 520 mm (W) × 155 mm (L) × 410 mm (H).

The device was tested at its maximum air flow value (300 m^3^ h^−1^).

### 4.2. Test System and Experimental Setup

**Figure 3:**
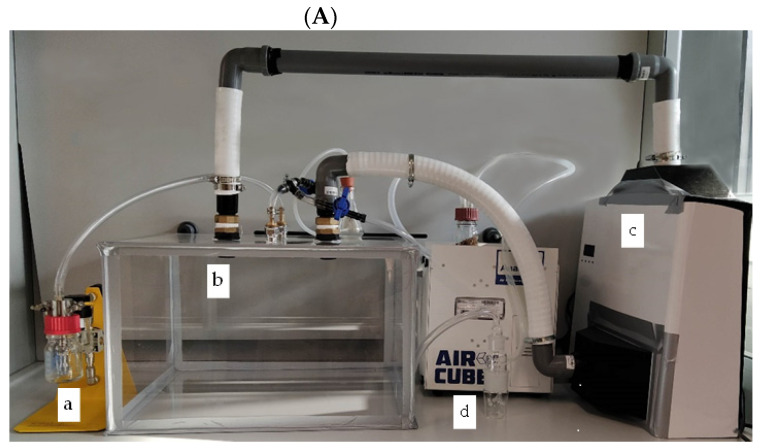

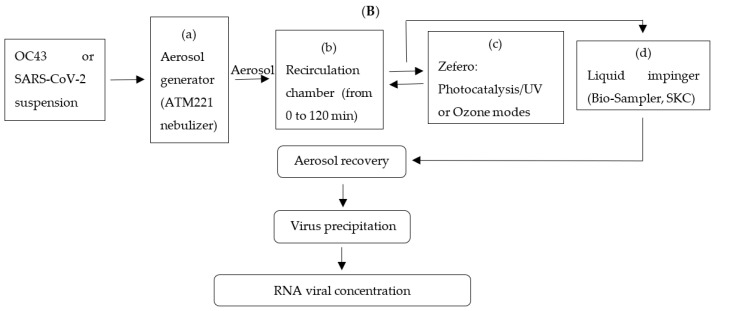
(**A**) Zefero device and the designed laboratory test system: (a) aerosol generator, (b) recirculation chamber, (c) air sanitizer Zefero and (d) liquid impinger. (**B**) Flow diagram of the experimental setup.

In Figure 3, the laboratory test system (Figure 3A) and the experimental setup (Figure 3B) used in this study are reported. The viral aerosol generated by an ATM221 nebulizer (Topas GmbH, Dresden, Germany), using compressed air at an operating pressure of 1.5 bar (a), was injected into a recirculation plexiglass chamber (b), with dimensions 500 mm (W) × 450 mm (L) × 300 mm and 67.5 dm^3^ in volume. The top of the chamber was connected to the air sanitizer Zefero (c) by tubes (40 mm diameter) with 3D printed adapters sealed to the front and upper grilles (device air input and output, respectively) to recirculate the aerosol. The top of the chamber also had an inlet for aerosol entry and a two-way output for air exhaust (connected to a 0.22 µm filter) or aerosol recovery by liquid impinger (Bio-Sampler, SKC Inc., Nottingham Township, PA, USA) (d). A vacuum pump (AirCube COM2, AMS Analitica, s.r.l., Pesaro, Italy) was used at a flow rate of 10 L min^−1^ for 30 min. The total volume of the laboratory test system, encompassing the plexiglass chamber, tubes and air sanitizer, was 86 dm^3^.

### 4.3. Betacoronavirus OC43 and SARS-CoV-2

Human Betacoronavirus OC43 ATCC VR-1558™ (ATCC, Manassas, VA, USA) was propagated on human colorectal ileocecal adenocarcinoma HCT-8 cells (ATCC: CCL-244), cultured in RPMI 1640 + 2 mM Glutamine + 1 mM Sodium Pyruvate + 10% Horse Serum + 100 U ml^−1^ penicillin, 100 μg mL^−1^ of streptomycin (Gibco, Grand Island, NY, USA). Infection was performed at MOI 0.01–0.1 on a cell monolayer of 18–48 h at 80–90% confluence. After 4–6 days, the cells were lysed by 2 cycles of freezing and thawing, then centrifuged at 900× *g* for 5 min at 4 °C to remove cell debris. The supernatant was collected, and the virus was titrated according to the Reed and Muench formula [50]. As a control, mock-infected HCT-8 cells were used. Virus and mock aliquots were stored at −80 °C until further use. Before use, OC43 aliquots were diluted to 1.5 × 10^4^ PFU/mL and thermally inactivated at 75 °C for 30 min in a thermostatic bath.

Inactivated SARS-CoV-2 virus from Amplirun^®^ Total SARS-CoV-2 Control kit (Vircell Molecular, Granada, Spain) was used for safety reasons. Lyophilized samples containing 1.2–2 × 10^4^ Genome Copy Equivalent (GCE) were reconstituted in sterile RNAse free H_2_O, following the manufacturer’s instructions. For each lyophilized sample, the SARS-CoV-2 titer was estimated as GCE by qRT-PCR and eventually normalized to a final concentration of 1.5 × 10^4^ GCE.

All media, reagents and glassware used for the preparation of viruses were initially sterilized by autoclaving at 121 °C for 15 min. All chemicals were from Sigma-Aldrich (St. Louis, MO, USA) unless otherwise specified.

### 4.4. Aerosol Generation and Recovery, Viral RNA Extraction and Quantification

#### 4.4.1. Aerosol Generation

Viral suspensions (5 mL) containing 1.5 × 10^4^ (GCE) of OC43 or SARS-CoV-2 viruses were used to generate polydispersed aerosols with particles from 0.3 to 5 µm in diameter.

#### 4.4.2. Aerosol Recovery and Virus Precipitation

After different times of exposure (15, 30, 45, 60, 75, 90, 105 and 120 min), samples were collected by a liquid impinger containing 5 mL of sterile RNAse-free H_2_O and concentrated by precipitation, according to the protocol described by IDEXX Laboratories [51], with some modifications. Briefly, PEG8000 (10% *w*/*v*) and NaCl (2.25% *w*/*v*) were added to the collected sample, and then the sample was centrifuged at 12,000× *g* for 2 h at 4 °C (Hermle Z 366 K, Hermle AG, Gosheim, Germany). Once the supernatant was discarded, the pellet was resuspended in 100 µL of sterile RNAse-free H_2_O and kept in an ice bath until further analyses.

#### 4.4.3. Viral RNA Extraction and Quantification

Viral RNA was extracted using the TRIzol Reagent kit (Invitrogen, Carlsbad, CA, USA) and reverse transcribed by ImProm-II™ Reverse Transcriptase kit (Promega Italia, Milan, Italy), according to the manufacturers’ instructions. Reverse transcription was performed in a 20 µL reaction mix containing 1 µg of total extracted RNA, 1× reaction buffer, 0.5 mM dNTP, 20 pmol primers, 3 mM MgCl_2_, 20 U RNase inhibitor and 200 U reverse transcriptase Improm II (Promega Italia). The reaction was carried out as follows: 25 °C for 5 min, 37 °C for 60 min and 70 °C for 15 min. cDNA samples were quantified by qRT-PCR.

For the relative quantification of OC43 viral genomic copies, the N gene was amplified using Sense 5′-AGGAAGGTCTGCTCCTAATTC-3′ and Antisense 5′-TGCAAAGATGGGGAACTGTGGG-3′ primers [52]. An amount of 5 µL of cDNA was used in PCR using 20 pmol of primers and 10 µL of SsoAdvanced Universal SYBR Green SuperMix (BioRad, Hercules, CA, USA). The sample was denatured at 95 °C for 5 min and amplified for 45 cycles (95 °C, 15 s; 58 °C, 30 s; 72 °C, 30 s).

SARS-CoV-2 Nucleic Acid Detection Kit (Hangzhou Bioer Technology Co., Ltd., Hangzhou, China) was used for SARS-CoV-2 quantization by amplification of regions within the N and ORF1ab genes. An amount of 5 µL of cDNA was added to 12.5 µL 2× RT-PCR Buffer, 1.3 µL of Enzyme mix and 6.2 µL 2019nCoV Primer/Probe. The reaction steps were as follows: 50 °C for 10 min, 95 °C for 1 min and 45 cycles of 95 °C for 10 s and 60 °C for 30 s. The reaction was carried out in a QuantStudioDx Real-Time PCR instrument Applied Biosystems Life Technologies thermal cycler, using the reading channels for FAM and VIC.

**Figure 4:**
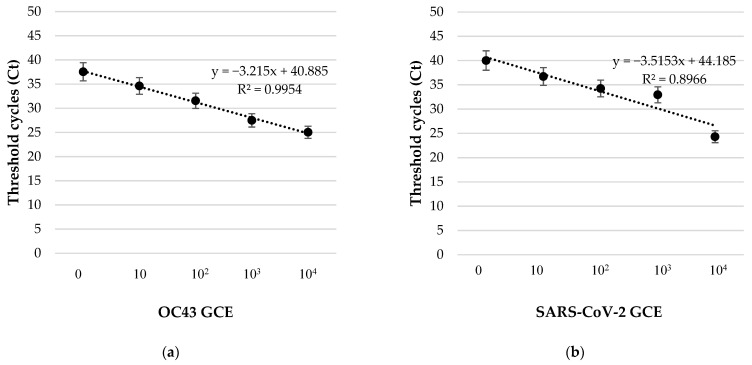
Standard curve of OC43 (**a**) and SARS-CoV-2 (**b**) genomic RNA, expressed as Genome Copy Equivalents (GCE) vs. Ct values.

In order to quantify the OC43 or SARS-CoV-2 viral copies in the aerosols, the standard curves were generated (Figure 4).

### 4.5. Evaluation of Viral Concentration Reduction (VCR)

After the exposure to each treatment (Photocatalysis/UV or Ozone mode), the viral concentration reduction (VCR), expressed as a percentage, was determined at each time (0, 15, 30, 45, 60, 75, 90, 105 and 120 min) as follows:

(1) $$VCR\left(\%\right)=100-\frac{C_{0}-C_{N}}{C_{0}}\times 100$$

where *C*_0_ is the viral concentration at time 0, and *C_N_* is the viral concentration after the treatment at time *N*.

### 4.6. Calculation of Half-Life Time

The time needed to halve the genomic RNA concentration of OC43 or SARS-CoV-2 after each treatment (half-life time, *t*_1/2_) was calculated according to the following formula:

(2) $$t_{1/2}=\frac{\ln2}{k}$$

### 4.7. Statistical Analysis

Data presented are an average of three independent experiments, and the error bars represent the standard deviation across independent experiments.

Regression analysis was performed using Prism 8.0 software (Graphpad Inc., San Diego, CA, USA).

## Figures and Tables

**Table 1:** Viral copies’ reduction in OC43, expressed as percentage (±SD), by Zefero device operating in Photocatalysis/UV or Ozone mode.

Time of Exposure (min)	Volume of Recirculated Air (m^3^)	Viral Copies Reduction (%) (±SD)	
		Photocatalysis/UV Mode	Ozone Mode
0	0	0 (±5.00)	0 (±4.76)
15	75	67.86 (±4.79)	90.20 (±4.63)
30	150	84.00 (±4.56)	100
45	25	92.00 (±4.00)	-
60	300	95.50 (±3.18)	-
75	375	97.90 (±3.49)	-
90	450	99.00 (±4.00)	-
105	525	99.50 (±1.20)	-
120	600	100	-

**Table 2:** Viral copies’ reduction in SARS-CoV-2, expressed as percentage (±SD), by Zefero device operating in Photocatalysis/UV or Ozone mode.

Time of Exposure (min)	Volume of Recirculated Air (m^3^)	Viral Copies’ Reduction (%) (±SD)	
		Photocatalysis/UV Mode	Ozone Mode
0	0	0 (±3.87)	0 (±4.65)
15	75	60 (±3.88)	60 (±4.68)
30	150	75 (±4.02)	85 (±4.13)
45	225	90 (±4.53)	100
60	300	95 (±4.26)	
75	375	99 (±4.00)	
90	450	100	

## Data Availability

The data supporting the findings of this study are available within this article Nicolò et al. Pathogens.
